# Clinical Significance of Extracellular Vesicles in Prostate and Renal Cancer

**DOI:** 10.3390/ijms241914713

**Published:** 2023-09-28

**Authors:** Tzu-Yi Chen, Meredith Mihalopoulos, Laura Zuluaga, Jordan Rich, Teja Ganta, Reza Mehrazin, Che-Kai Tsao, Ash Tewari, Edgar Gonzalez-Kozlova, Ketan Badani, Navneet Dogra, Natasha Kyprianou

**Affiliations:** 1 Department of Pathology & Cell Based Medicine, Icahn School of Medicine at Mount Sinai, New York, NY 10029, USA; tina.chen@icahn.mssm.edu (T.-Y.C.); ash.tewari@mountsinai.org (A.T.); 2 Department of Urology, Icahn School of Medicine at Mount Sinai, New York, NY 10029, USA; meredith.mihalopoulos@icahn.mssm.edu (M.M.); laura.zuluaga@mountsinai.org (L.Z.); jordan.rich@mountsinai.org (J.R.); reza.mehrazin@mountsinai.org (R.M.); ketan.badani@mountsinai.org (K.B.); 3 Department of Hematology/Oncology, Icahn School of Medicine at Mount Sinai, New York, NY 10029, USA; teja.ganta@mountsinai.org (T.G.); che-kai.tsao@mssm.edu (C.-K.T.); 4 Department of Oncological Sciences, Icahn School of Medicine at Mount Sinai, New York, NY 10029, USA; edgar.gonzalez-kozlova@mssm.edu; 5 The Tisch Cancer Institute, Mount Sinai Health, New York, NY 10029, USA

**Keywords:** exosomes, liquid biopsies, therapeutic targeting, prediction, tumor progression

## Abstract

Extracellular vesicles (EVs)—including apoptotic bodies, microvesicles, and exosomes—are released by almost all cell types and contain molecular footprints from their cell of origin, including lipids, proteins, metabolites, RNA, and DNA. They have been successfully isolated from blood, urine, semen, and other body fluids. In this review, we discuss the current understanding of the predictive value of EVs in prostate and renal cancer. We also describe the findings supporting the use of EVs from liquid biopsies in stratifying high-risk prostate/kidney cancer and advanced disease, such as castration-resistant (CRPC) and neuroendocrine prostate cancer (NEPC) as well as metastatic renal cell carcinoma (RCC). Assays based on EVs isolated from urine and blood have the potential to serve as highly sensitive diagnostic studies as well as predictive measures of tumor recurrence in patients with prostate and renal cancers. Overall, we discuss the biogenesis, isolation, liquid-biopsy, and therapeutic applications of EVs in CRPC, NEPC, and RCC.

## 1. Extracellular Vesicles—Introduction

Almost all types of cells, including cancer cells, release a diversity of extracellular vesicles and particles [1,2], comprising of apoptotic bodies (800–5000 nm) [3,4], microvesicles (50–2000 nm) [5,6], exosomes (~30–200 nm) [7,8], and other nanoparticles [9,10,11,12]. EVs contain molecular footprints from their cell of origin [2], including lipids, proteins, metabolites, RNA, and DNA [6,7,8,9,10,11,13,14]. They are present in almost all types of cells and have been successfully isolated from blood, urine, semen, and other body fluids [15,16,17]. EVs have been widely studied for their role in the tumor microenvironment (TME) and cancer progression and metastasis, specifically their involvement in cell-to-cell communication via vesicle trafficking, molecular targeting and intricate delivery mechanism [4,12,18,19]. These characteristics and utilities lay the foundation for EVs as potential biomarkers for the diagnosis and prognosis of prostate cancer [20]. Reports of prostatic and seminal fluid-derived EVs were recorded as early as the 1980s [21,22,23,24] and were demonstrated to contain the highest concentration of prostate-derived EVs [25]. Nevertheless, the majority of prostate cancer EV studies [26,27,28,29] are focused on investigating the proteins and miRNAs in EVs from patient urine or blood given their accessibility and non-invasive nature [20,30]. 

In the early phases of EV research (in the 1980s), EVs were originally thought of as cargos of cell debris and wastes [27]. Nevertheless, beginning in 1990s, studies showed results suggesting EVs’ pivotal role in cell-to-cell communications and as triggers for cancer immune responses [28,29]. Major breakthroughs were marked in the 2000s, as mRNAs and microRNAs were unveiled in EVs along with their influence on cellular behaviors and functions [13,31]. In particular, a wide variety of genetic materials were gradually identified, including mRNA, ncRNA, miRNA, lncRNA, ssDNA, dsDNA, mitochondrial DNA, and oncogene amplifications [2,32,33,34,35,36]. In addition to genetic material, EVs also deliver lipids and proteins [37,38]. The inclusion of proteomic components and genetic materials suggests that exosomes have the capability of regulating and triggering specific signaling cascades and thereby altering the transcriptional landscape of the targeted cell [2]. These characteristics identify EVs as key modulators in disease progression to metastasis, defining the tumor microenvironment, mainly through cellular crosstalk and vesicle trafficking [37].

**Figure 1:**
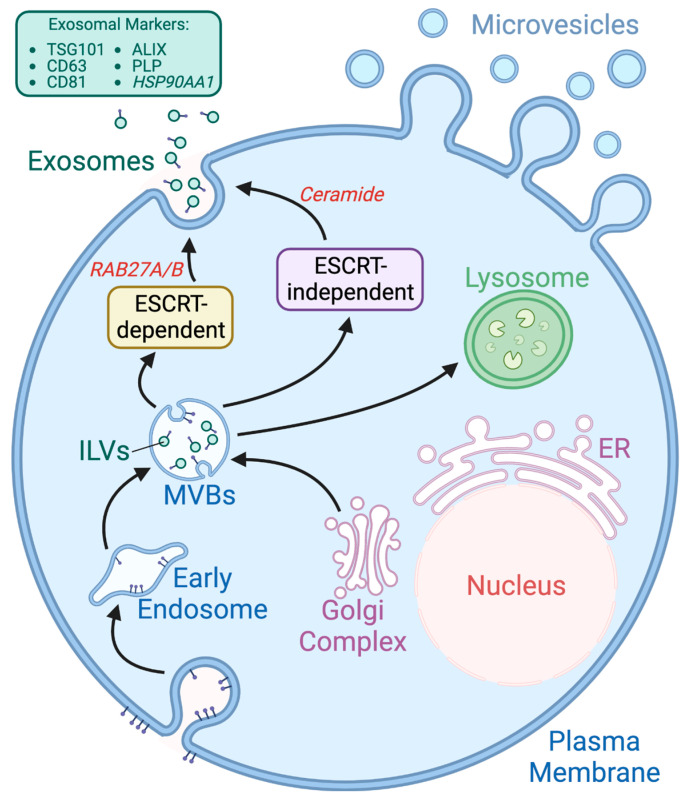
Overview of EV Biogenesis. Exosomes are initially intraluminal vesicles (ILVs) that are formed via inward budding of multivesicular bodies (MVBs). The MVBs then fuse with the plasma membrane to release the exosomes. Both the ESCRT-dependent pathway (uses RAB GTPase proteins) and the ESCRT-independent pathway (uses ceramide) are associated with the regulation of exosome secretion [48].

Exosomes are formed by inward budding of the peripheral membrane of late-stage endosomes, otherwise known as multivesicular bodies (MVBs) [4,39]. Sequentially, the MVBs perform the extracellular release of exosomes by fusing with the plasma membrane (Figure 1) [4,18]. Recent studies have indicated that the classification of exosomes is largely dependent on the given intracellular trafficking pathways, resulting in the different vesicle sizes and cargo contents [37,38,40,41]. The endosomal sorting complexes required for transport (ESCRT) proteins along with the Rab small GTPase family serve a crucial role in modulation of exosomal secretion and trafficking [27]. The mechanism starts with the ESCRT-0 protein utilizing hepatocyte growth factor-regulated tyrosine kinase substrate (HRS) to identify and cluster ubiquitinated transmembrane proteins in the endosomal membrane. Once properly localized, the HRS recruits ESCRT-I/II complexes along with associated proteins, for instance, TSG101, ALIX, VPS4, etc., for initiation of MVBs biogenesis via budding. Finally, the actual process involving vesicle scission is primarily driven by the ESCRT-III protein. Free ESCRT components and ubiquitin molecules are recycled for repeating the post-scission process of the MVBs [27,38]. Following the formation of MVBs, the remainder of the trafficking pathways comprised of cytoskeleton, molecular motors, and vesicle fusion machinery are mostly regulated by the RAB family of small GTPases [42]. Gene knockouts of RAB2B, RAB9A, RAB5A, RAB27A or RAB27B are shown to be correlated with effective inhibition of exosome biogenesis [42,43,44]. In particular, both RAB27A and RAB27B are associated with promotion of MVBs docking and fusing to the plasma membrane, as well as the vesicle transfer from the Golgi to MVBs. Likewise, mechanisms involving RAB small GTPases often recruit SNAP receptors (SNAREs), a superfamily of proteins, for mediation of vesicle trafficking within the cells [42,43]. Despite the critical role of the ESCRT complexes, alternative ESCRT-independent pathways of exosomal packaging and formation have also been shown [43]. Certain molecules that do not require ubiquitination, including proteolipid protein (PLP), are observed in exosomal cargoes via sphingolipid ceramide. Trajkovic et al. first discovered the cargo segregating mechanism, involving sphingomyelinases enriched raft-based microdomain found in oligodendroglial cell lines [45]. Sphingomyelinases would actively promote the hydrolysis of sphingomyelin into ceramides, known to induce spontaneous negative curvatures and domain-induced budding. Consequently, the ESCRT-independent process emphasizes the extensive role of ceramide and lipids in exosome biogenesis [46,47]. In addition to proteins actively involved in exosomal biogenesis (i.e., TSG101, ALIX, RAB proteins, and annexins), other frequently observed exosomal proteins include membrane transport proteins, metabolic enzymes, fusogenic proteins, tetraspanins, heat shock proteins, cytoskeletal proteins (e.g., actin and tubulin), lipoproteins, and enzymes (e.g., phospholipases). Differences across cell types and phenotypes are also reflected across proteomic profiles of corresponding exosomes excreted [2,42].

## 2. Prostate Cancer

### 2.1. Introduction

With an incidence of 268,000 cases in the United States in 2022 [49], prostate cancer is the second most commonly diagnosed solid tumor in men and the fourth most common cancer across all sexes [50]. There were over 34,500 deaths in the US in 2022 [49], which ranks prostate cancer as the fifth leading cause of cancer mortality in men [50]. Currently, the diagnosis of prostate cancer is based on serum prostate-specific antigen (PSA) levels, digital rectal examination (DRE), and, if indicated, biopsies guided by transrectal ultrasonography (TRUS). While most guidelines incorporate PSA as a biomarker, it has a low positive predictive value (~30%) and poor specificity in prostate cancer diagnosis [51]. This often leads to a higher number of unnecessary biopsies and detection of asymptomatic cancers with low clinical risk [19]. It is critical to continue to further our understanding of non-invasive techniques in order to minimize the use of TRUS biopsies and prevent overtreatment of clinically low-risk patients. This has led to intense investigations of liquid biopsy biomarkers derived from extracellular vesicles and particles for both prostate cancer detection and profiling of cancer pathogenesis. Such tools include prostate cancer antigen 3 (PCA3), circulating tumor cells (CTCs), extracellular vesicles (EVs), circulating tumor DNA (ctDNA) and RNA (ctRNA), and genetic biomarkers like TMPRSS:ERG gene fusions [52] (A detailed list of prostate cancer derived EV markers is provided in Table 1). Other urine biomarkers developed are Mi-Prostate Score (MiPS), ExoDx Prostate (IntelliScore) (EPI), SelectMDx Prostate Health Index (PHI) and 4KScore, while ConfirmMDx (MDxHealth) is a tissue biomarker utilized in some settings [19]. 

Prostate cancer development requires androgen receptor (AR)-mediated signaling, which makes androgen deprivation therapy (ADT) the standard first-line treatment for patients with advanced disease [81,82]. Through gene mutation and amplification, prostate cancer cells are able to develop resistance to ADT treatment by restoring AR signaling, ultimately yielding castration-resistant prostate cancers (CRPC) [18]. Given that CRPC tumors remain AR-dependent, they are treated with potent AR pathway inhibitors (ARPIs), such as abiraterone, enzalutamide, apalutamide and darolutamide [18]. Certain cancers are able to perpetuate AR-independent mechanisms and develop resistance to ARPIs—this subset of CRPC cases is referred to as neuroendocrine prostate cancer (NEPC). These tumors are characterized by a low level or lack of AR expression, an independence of AR signaling, and a gain of neuroendocrine phenotype [83,84], often becoming AR-negative, poorly differentiated small cell neuroendocrine carcinoma [13,14]. In this review, we focus on the use of EVs as a promising tool for diagnosis of patients with prostate cancer, particularly those with advanced NEPC.

### 2.2. Role of EVs in Prostate Cancer Progression

EVs have been found to be more abundant in prostate cancer patients than in healthy individuals and are secreted in higher quantities by prostate cancer cells as compared to normal prostate cells [26,32,33,34,35,36,37]. Patients with prostate cancer have been found to have a four-fold higher level of nanovesicles expressing PSA and CD81 as compared to healthy men or those with benign prostate hypertrophy (BPH) [15,38]. Logozzi et al. proposed that TME acidity may regulate the release of PSA-EVs in the blood of patients with prostate cancer [38]. These findings demonstrate the potential use of EVs from liquid biopsies in stratifying high-risk prostate cancer. McKiernan et al. designed the ExoDx Prostate (IntelliScore) urine exosome gene expression assay, which quantifies the expression of three genes (PCA3, ERG, and SPDEF) from urine in patients with equivocal PSA level [40] and remains the only exosome-based liquid biopsy successful assay approved by the FDA for any malignancy thus far [41,42]. The use of exosomal assays can help in preventing overdiagnosis and overtreatment in order to better assess which patients benefit from aggressive interventions. EVs isolated from urine of patients have shown the use of this assay as a means of diagnosing cancer with high sensitivity and predict tumor aggressiveness and recurrence after radical prostatectomy and radiotherapy [27,43]. Moreover, EVs have been shown to play a pivotal role in promoting metastases of prostate cancer by establishing the pre-metastatic niche (PMN) [32], another important attribute for proper diagnosis and staging of the cancer. Growing evidence suggests that metastatic progression is mediated by exosomal microRNAs, short non-coding RNA sequences that can regulate post-transcriptional gene expression [44]. Aberrant expression of various specific miRNAs has been observed in several tumors, including breast [46], lung [47], colorectal cancer [85], ovarian [86], and prostate cancer [16,87]. Exosomal miRNA has indeed served as a potential useful biomarker in several human malignancies for better distinguishing tumor tissue from normal tissue, classifying tumor origin, and understanding tumor staging [16,88]. Mechanistically, microRNAs mediate the crosstalk between tumor cells and the TME to regulate tumor growth [89]. These exosomal miRNAs affect cancer progression by reducing apoptosis, increasing proliferation, migration, adhesion of cancer cells [90,91,92], and regulating the phenotypic epithelial–mesenchymal transition (EMT) of prostate cancer cells [93]. These miRNAs also contribute to ADT resistance by creating a TME enabling tumor cell proliferation, differentiation, and angiogenesis and recruiting fibroblasts and immune cells [16].

Several clinical studies have investigated the value of certain EV miRNAs in determining prostate cancer aggressiveness. Barceló et al. analyzed 400 different miRNAs from semen samples of patients with BPH, healthy controls, and patients with moderately elevated PSA levels and Gleason Score (GS) 6–8 in order to find a reliable biomarker for early diagnosis of prostate cancer [53]. The authors found the most diagnostic potential from the combination of PSA serum level, miR-142-3p, miR-142-5p, and miR-223-3p, which demonstrated a sensitivity of 91.7% and specificity of 42.9% [53]. Further, independent studies utilized semen samples to investigate cancer aggressiveness through cytokine tumor necrosis factor-like weak inducer of apoptosis (TWEAK)-regulated exosomal miRNAs, ultimately finding that with miR-221-3p, miR-222-3p, and TWEAK, it was possible to classify prostate cancer in terms of its aggressiveness with specificity at 85.7% and sensitivity at 76.9% [94]. Analysis of blood plasma samples of prostate cancer patients (26 with GS ≤ 6 and 24 with GS ≥ 8), revealed that the exosomal expression of miR-let-7a-5p was significantly lower in patients with a higher GS (≥8), compared to lower GS (≤6) [95]. However, there is controversy surrounding correlation as other studies found that the urinary exosome profiles of miR-let-7a-5p are not significantly different between low-risk and high-risk patients or between patients with metastatic CRPC and localized prostate cancer [96,97]. Urine samples did not prove to be of diagnostic value in terms of differentiating between prostate cancer patients and healthy controls; however, the expression of miR-1246 had a specificity of 100% and sensitivity of 75% in predicting metastases [98]. EV miR-4287 also had a specificity of 88.24% in predicting possibility of metastasis in early-stage prostate cancer [99]. Work from our group demonstrated that tumor-derived EVs (tdEVs) expressed altered levels of reactive oxygen species (ROS), P53 pathways, inflammatory/cytokines, oncogenes, and tumor suppressor genes in the EV nanosatellites after tumor resection [26].

### 2.3. EVs in Castration-Resistant Prostate Cancer (CRPC)

The current understanding of the use of EVs for the diagnosis and prognosis of prostate cancer continues to evolve as we further assess the value of exosomes and miRNA. There have been recent investigations focused on the role of EVs in prostate tumor progression to advanced aggressive disease and the emergence of castration-resistant prostate cancer (CRPC). In a study evaluating six different exosomal miRNAs, Guo et al. demonstrated that miR-423-3p was associated with the development of CRPC [54]. Others have found that the basis of this may be that EVs derived from mesenchymal-like prostate cells promote epithelial-mesenchymal transition (EMT) of epithelial-like prostate cancer cells and render resistance to androgen-deprivation therapy (ADT) [100]. miR-34a bearing EVs were suggested as a predictive biomarker since it was observed to promote sensitivity to docetaxel by decreasing endogenous B-cell Lymphoma 2 (BCL- 2) expression [55]. These tumor-derived EVs (tdEVs) are important, as they are also known to regulate osteoclast and osteoblast in the bone metastasis of prostate cancer patients [12]. Copies of miRNAs, such as CD44v8-10 mRNA, have been found in higher numbers in EVs in docetaxel-resistant CRPC patients than in docetaxel naive patients and control men [56].

Moreover, efforts have been directed to the detection of exosomal miRNAs associated with AR and distinguishing CRPC from NEPC. Using plasma EVs in CRPC patients, Joncas et al. discovered a novel association between high levels of AR-V7 exosomal mRNA (with undetectable androgen levels) and high neutrophil-to-lymphocyte ratio [57]. High expression of full-length androgen receptor (AR-FL) was also linked with AR-V7+ CRPC patients and predicted resistance to hormonal therapy [101,102]. The combination of several miRNAs in liquid biopsies provides promise for a prognostic signature. Huang and colleagues used RNA sequencing to interrogate the association of miR-1290 and miR-375 and overall survival (OS) rates of patients with CRPC [58]. High levels of plasma exosomal miR-1290 and miR-375 were associated with significantly worse OS (7.2 months vs 19.3 months, *p* = 0.0045) in a cohort of 100 CRPC patients. This study revealed that patients with high concentrations of both miRNAs had overall mortality rates (around 80%), while patients with average or low concentrations of both miRNAs had a considerably low mortality rate (10%) over the same 20-month follow-up period [58]. In a similar pattern, exosomal TUBB3 mRNA positivity and greater number of copies were associated with poor PSA progression-free survival (PFS) in patients with metastatic CRPC treated with abiraterone (positivity 7.9 months vs no TUBB3 11.0 months, *p* = 0.014) [59]. While AR-V7 has predominantly been detected through circulating tumor cells (CTCs), Del Re et al. achieved detection using the Droplet Digital PCR (ddPCR) system from plasma-derived exosomal RNA of mCRPC patients (n = 36) [103]. In this cohort, 14 patients who had received either enzalutamide or abiraterone had AR-V7 positivity and experienced both decreased median PFS (3 months vs 20 months, *p* < 0.001) and median OS (8 months vs NR, *p* < 0.001) [103]. This detection method potentially provides a simpler, less intensive, and cheaper method of detecting AR-V7 status compared to using CTCs without the limitations of CTCs (as molecular heterogeneity) [41,103,104]. Nanou et al. utilized the CellSearch system in the blood of CRPC patients and enumerated several subclasses of CTCs and tdEVs, finding that patients with >5 CTCs and >10^5^ tdEVs were associated with poor OS [105]. The study also showed that tdEVs improved predictive power, sensitivity, and specificity, when compared to CTC count alone [105,106]. Despite the smaller sizes of tdEVs, the investigators argue that tdEVs confer better stability and greater tumor heterogeneity, which makes it a more promising biomarker than CTCs in metastatic disease [15]. These data highlight the importance of exosomal mRNA and EVs in the diagnosis and prognosis of aggressive prostate cancer, as well as the need for further investigation on a larger scale for such use of liquid biopsies. To establish the diagnostic capability of EVs, work from our group has devised a comprehensive diagnostic model using pre-established prostatic EV markers found in prostate cancer cohorts. Dogra et al. have validated 12 EV markers that could effectively discern pre- and post-tumor resection conditions via differential expression analysis of over 60 total small RNAseq profiles from 17 aggressive prostate cancer patients and their matching adjacent normal tissue, serum, and urine EVs [26]. Of the twelve markers, genes differentially expressed in pre-resection EVs include NKX3-1, BRCA1, MXD4, CYLD, IRF1 ESR1, SMYD3, FOXO3, and HAS2; genes differentially expressed in post-resection EVs include SNORA54, UPK1B, and TET3 [26]. Nevertheless, the most intriguing finding is the upregulation of major tumor suppressors (NKX3-1, BRCA1, MXD4) in the EVs of the pre-resection prostate cancer patients instead of in those of the cancer free patients [107]. The result of the pathway enrichment analysis suggests these EV markers are largely associated with signaling pathways, including miRNA, Wnt signaling, T-cell receptor, hormonal, cytokines, and growth factor related pathways. Of these, over 80% of the PCa serum EV samples are enriched in non-canonical Wnt Signaling (*p* = 0.0039), emphasizing EVs’ role in the Wnt signaling-associated seeding and dissemination mechanism. As a result, Dogra et al. have concluded this is an explicit representation of EVs’ unique and selective package mechanism for the distribution of tumor-associated genetic materials [26]. 

**Figure 2:**
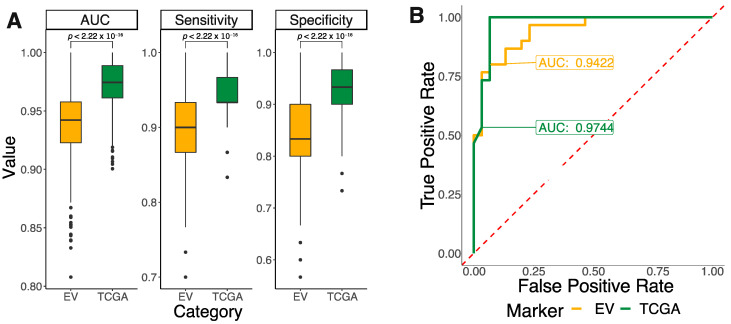
Performances of the model based on the 12 prostate EV markers vs. the one based on top TCGA differentially expressed markers. (**A**) AUC (measurement of how well the model separates the two groups and predicts the correct class), sensitivity (measurement of ability to predict true positives), and specificity (measurement of ability to predict true negatives) of the two models. (**B**) Receiver Operating Characteristics curve visualizes model performances at different classification thresholds. The area underneath the curve is the AUC.

Consequently, we leveraged the Prostate Adenocarcinoma cohort of The Cancer Genome Atlas (TCGA-PRAD), comprised of over 497 primary tumor samples and 52 adjacent normal tissue, to train and test diagnostic models using either these 12 EV markers or the top differentially expressed markers of the TCGA-PRAD cohort. The models utilize the random forest algorithm, a supervised learning algorithm that functions by aggregating the results of multiple decision trees [108,109]. To effectively limit overfitting and number of errors due to bias, we implemented bootstrap resampling (n = 30), a random sampling statistical method with replacement, to output the same model performance indices. Likewise, to provide a more stringent estimate of model accuracy, cross validation is performed 1000 times. The results of the model, as measured by area under the curve (AUC), sensitivity, and specificity, suggested that the model using differentially expressed TCGA markers has better performance than that using the EV markers (AUC: 97%, sensitivity: 95%, specificity: 0.92%). (Figure 2). Regardless, given that this is not the original dataset, the classification performances of the model based on EV markers still showed high accuracy (AUC: 94%, sensitivity: 89%, specificity: 84%). This is a clear representation of the capability of EV as a surveillance tool for early prostate cancer detection via non-invasive liquid biopsy. Paired with pre-validated EV markers, liquid biopsy diagnostic models for different cancers would be highly probable in a clinical setting. 

### 2.4. EVs in Neuroendocrine Prostate Cancer

It is important to specifically review EVs in NEPC, given the lethal nature of the disease. Bhagirath et al. performed small RNA next generation sequencing in serum EVs isolated from a cohort of CRPC patients, comparing those with adenocarcinoma characteristics (CRPC-Adeno) and neuroendocrine characteristics (CRPC-NE) [60]. The authors identified significant dysregulation of 182 known and 4 novel miRNAs. Utilizing a machine learning algorithm to develop an “EV-miRNA classifier” that could robustly stratify “CRPC-NE” from “CRPC-Adeno”, the authors identified thrombospondin 1 (TSP1) as a specific biomarker in the exosomes of their NEPC cellular models and proposed this as a potential novel EV biomarker for detecting NEPC in advanced castration-resistant prostate cancer patients [60].

Several miRNAs have been isolated as regulators of molecules associated with the NED phenotype. These usually include increased expression or dysregulation of miR-375, miR-34a, miR-19b-3p, and miR-30d-5p [60]. mi-R-375 in particular has been a focus of study for its diagnostic and prognostic potential in distinguishing benign and aggressive diseases as well as predicting treatment response [110,111]. Certain miRNAs, such as miR-301a, miR-34a, and miR-30 family members, were identified as AR regulators with binding sites in both UTR and coding regions of AR. Specifically, the loss of miR-30c-5p and miR-30d-5p expression correlated with advanced disease [112]. Others have studied novel transcription factors (TFs) BRN4 and BRN2 in EVs that drive oncogenic reprogramming of prostate cancer cells to the NEPC phenotype; these EV-associated TFs may then serve as important non-invasive biomarkers in predicting NED in CRPC [61]. EVs may also play a role in driving lineage plasticity by reprogramming cells. For instance, prostate cancer cells expressing integrin αVβ3 release EVs that are capable of stimulating tumor growth and driving neuroendocrine differentiation in pre-clinical models [113,114]. Considering the production of hormones and growth factors from neuroendocrine cells, there is the potential of hormones such as growth hormone-releasing hormone (GHRH) to influence NED, as seen in androgen-dependent prostate cancer cell lines; simulation by GHRH involves calcium channel activation and EGFR/HER2 transactivation [115]. While the application of EVs is unclear in this hormonal influence, studies of GHRH-induced stimulation of NED revealed that PC3-derived EVs increased cell differentiation, proliferation, and adhesion [115]. The impact of hormones in NEPC must also be considered in exploitation of EVs in the context of the endocrine milieu of the TME [116]. 

## 3. Renal Cancer

### 3.1. Introduction

Cancers of the kidney represent about 4% of all new diagnosed cancers, with more than 75,000 Americans diagnosed and about 14,000 dying due to the disease in 2021 alone [49,117]. About 90% of these cancers are renal cell carcinoma (RCC), and about 70% of all renal cancers have a clear cell histology (ccRCC), with other less common histology including papillary and chromophobe [117,118,119,120]. Although some patients only present in the advanced stage of the disease with systemic symptoms or symptomatic pain, most cases are detected incidentally with imaging for the workup of other abdominal diseases or nonspecific symptoms, a phenomenon that is continually increasing with the rising use of medical imaging and subsequence incidence of RCC [121,122,123]. The well-described triad of RCC, including flank pain, hematuria, and a palpable mass is very rarely identified and represents very aggressive disease [122]. Imaging, specifically CT and MRI, helps distinguish RCC from cystic lesions [124], but the procedure is not without pitfalls, as CT and MRI cannot reliably distinguish malignant kidney cancer from some benign angiomyolipoma (AML) and oncocytomas [122]. Diverse factors appear to be limiting the ability of these approaches to distinguish between benign and malignant lesions [125]. These also represent costly tests for the patients and the healthcare system at large, and CT scans introduce radiation to the patient [126]. Despite improvements and increased utilization of imaging modalities, one in every five patients is found to have metastasis at diagnosis which portends much poorer survival, suggesting there is an unmet clinical need for improved kidney cancer diagnostic technology [127].

Current diagnostic paradigms in kidney cancer are associated with morbidity, high cost, and overtreatment. The use of percutaneous kidney tumor biopsies is restricted, and guidelines recommend their use solely for small lesions to confirm malignancy and assist in surveillance or ablative techniques, differentiate between benign and malignant tumors, and identify patients suitable for active surveillance, cryosurgery, and radiofrequency ablation strategies [122]. However, biopsies are invasive with associated morbidity and questionable sensitivity and specificity for histologic diagnosis. Non-invasive methods contribute to the diagnosis of kidney cancers and may help facilitate early detection of aggressive tumors [128]. Recently, abdominal insufflation samples during laparoscopic surgery were analyzed non-invasively [129]. Furthermore, early screening and distinguishing between benign and malignant masses may help improve survival and avoid unnecessary surgery [74,122,130]. 

### 3.2. The Value of EVs in Kidney Cancer Detection and Diagnosis

EVs are utilized by tumor cells to transport bioactive molecules to other tumor cells and CAFs, cancer stem cells, immune cells, or endothelial cells [63,64], contributing to the complex tissue dynamic network surrounding the tumor. EVs secreted in the TME can contribute to RCC development and progression [65]. Most EV studies have focused on ccRCC [66] (A detailed list of renal cancer derived EV markers is provided in Table 1). One study found that high levels of a lncRNA in serum (LNARSR), a type of EV cargo in plasma, can differentiate patients with ccRCC from healthy controls [67]. Also in serum, one study found that the protein molecule Azurocidin was increased in ccRCC patients compared to healthy controls [68]. Azurocidin (AZU1), a molecule closely associated with angiogenesis and cell migration processes in RCC, is highly expressed in EVs derived from tumor cells. AZU1 from ccRCC EVs indeed plays a critical role in disrupting the morphology of vascular endothelial cells, resulting in increased cell transmigration. Furthermore, it suggests that AZU1-EVs produced by ccRCC cells may aid in the hematogenous metastasis of ccRCC cells and serve as metastasis promoters. Evidence suggests that EV-AZU1 could be a highly promising early detection biomarker for RCC, which could be detected through a non-invasive serological test [68]. Circulating miRNAs are promising diagnostic candidates due to our ability to isolate them from biologic liquids noninvasively. In urine, studies have found that the combination of microRNA miR-126-3p and miR-449a could be used to differentiate ccRCC from healthy controls; miR-30c-5p was found to be significantly lower in ccRCCC patients compared to healthy controls [69,70]. Zhang et al. found that exosomal miR-210 and exosomal miR-1233 were higher in each stage of ccRCC compared to healthy controls, and another study also found miR-210 was upregulated in ccRCC, lending credence to the possibility that this combination may be used for early ccRCC detection [71,72]. Song et al. found miR-30c-5p in urinary exosomes of ccRCC to be significantly lower than that in healthy control patients [69]. A few clinical trials have tested miRNA for RCC detection. Chen et al. used a panel of miR-21-5p, miR-150-5p, miR-145-5p, and miR-146a-5p, showing an AUC of 0.938 [131]. miR-21-5p, hypothesized to play a role in the epithelial mesenchymal transition, was also used as part of a four-miRNA urine panel, with a sensitivity of about 83% [132,133]. A few trials have used single miRNA panels as they are more cost effective and efficient. One small trial found that miR-15a in urine had an AUC of 0.955 (sensitivity of 100% and specificity of 98.1%) for differentiating RCC from benign renal tumors [134]. Additional investigative efforts have found singular circulating miRNAs markers without much concordance between studies [73,75,135,136,137,138,139]. Significantly enough, another study found polymerase 1 and transcriptase release factor (PTRF) to be higher in ccRCC patients compared to healthy controls and that it diminished after nephrectomy, suggesting its involvement in the kidney TME [140]. Moreover, application of high throughput sequencing detected five novel mRNAs specific for stage I ccRCC in urinary exosomes, including NME2, AAMP, CAPNS1, VAMP8, and MYL12B [66,141]. More studies and large sample sizes are required to identify the diagnostic capacity of these EV-derived biomarkers for RCC. 

Circulating tumor cells have been considered a sign and cause of tumor recurrence and metastasis [130,142]. Thus, they maintain less of a role for diagnosis than they do for progression and prognosis [130,142]. Additionally, ctDNA in RCC is found in lower levels than in other malignancies with low concordance with tissue genomic data [76,143]. Circulating free DNA (cfDNA) is used more often in RCC as results have been more promising. Recent studies have shown that DNA methylation analysis in cfDNA may be of use in RCC. One study showed that DNA methylation scores showed a sensitivity of 100% and specificity of 88% for RCC [144]. Nuzzo et al. also constructed a methylation scoring model using cfDNA, using it to accurately diagnose 67 of 69 patients with RCC, with an AUC of 0.99 [145]. Of note, the methylation of genes such as VHL, RNF185, or RASSF1A has been shown to generate ROC curves with AUCs between 0.694 and 0.755 for the diagnosis of RCC, with an increase in diagnostic accuracy to 100% using multiple methylation targets with cfDNA quantification [146]. Overall, RCC cfDNA levels have been investigated as well, with controversial results due sample size and similar technical limitations. Thus, while De Martino et al. found increased cfDNA levels in patients with RCC compared to benign kidney tumors and that cfDNA levels predict kidney malignancy with a AUC 0.755 [147], another study found no difference in the amount of most of the cfDNA fragments between RCC patients and healthy controls. There was a relative increase in the shorter DNA fragments in RCC patients, suggesting that those with cancer have more DNA fragmentation [148,149]. While advanced methodologies are constantly being developed, EVs, circulating tumor cells, and cfDNA provide a promising basis for the development of future diagnostic tools in kidney cancer. 

### 3.3. Role of Liquid Biopsy in Kidney Cancer Prognosis

The formation and evolution of cancer is significantly influenced by EVs and their functional involvement in intercellular communication within the TME. Studies comparing EVs from tumor cells and EVs derived from non-tumor cells have demonstrated the important role of these entities in intercellular communication, tumor progression to metastasis, and the landscape of the TME promoting dispersion of organ-confined disease [66]. The release of tumor-secreted factor (TSF) and tumor-secreted exosome (TSE) mediates the TME to adapt to become suitable for the migration of tumor cells in secondary organs [66,150]. This new TME component is called a pre-metastatic niche (PMN), and TSE is considered to be the primary driver of PMN formation. Tumor cells must take care of three critical aspects in order to be able to consolidate the PMN: 1. Angiogenesis/vascular permeability, 2. Epithelial-mesenchymal transition (EMT), and 3. Energy metabolism reprogramming [66] (Figure 2). 

### 3.4. EVs in Reprogramming the Tumor Microenvironment Phenotypic Landscape

Tumor-derived EVs influence tumor angiogenesis during tumor progression to metastasis, delivering pro-angiogenic factors to endothelial cells and modulating their behavior [151]. Additionally, exosomes have been found to promote tumor angiogenesis and metastasis, highlighting their potential as therapeutic targets in cancer [152]. 

Another important target of EVs is apolipoprotein C1 (ApoC1), a member of the apolipoprotein family, which has a significant impact on the metabolism of very low-density lipoproteins (VLDL) and high-density lipoproteins (HDL) [153,154]. It is becoming increasingly evident that ApoC1 is linked to the advancement of different tumors [154,155]. In a study by Li et al. focusing on ccRCC, the authors found that EV mediates the transfer of ApoC1 from ccRCC cells to vascular endothelial cells, which is related to angiogenesis facilitation by activating STAT3 and augmenting the migratory and invasive capacities of endothelial cells [153]. Highly invasive ccRCC cells exhibited higher levels of ApoC1 expression compared to the low-invasive ccRCC cells [153], potentially facilitating metastasis of ccRCC cells by inducing EMT, whereas reducing ApoC1 expression mitigated these effects [153]. Extensive descriptions exist of how tumor EVs promote angiogenesis by transporting pro-angiogenic mRNAs and miRNAs and modifying angiogenic pathways [156]. The following miRNAs have been found to interact with endothelial cells (thus promoting pro-angiogenic activity): miR-23a, miR-210, miR-135b, miR-494, miR-1246, miR-9, and miR-183/182/96 [157]. Specifically, miR-183/182/96, has a high importance in ccRCC due to its association with multiple clinicopathological features. The product of the miR-183/182/96 gene cluster regulates cell proliferation, migration, and tumor metastasis in different cancers [77]. According to a study conducted in 2018, high expression of miR-183/182/96 in RCC facilitates the proliferation and invasion of tumor cells by targeting Dickkopf-related protein-3 (DKK-3), which is a negative regulator of the Wnt signaling pathway [158]. An additional recent analysis of sera from 284 patients with RCC who underwent nephrectomy found a positive association between the level of miR-183 and a poor prognosis, suggesting its value as a prognostic biomarker [159].

**Figure 3:**
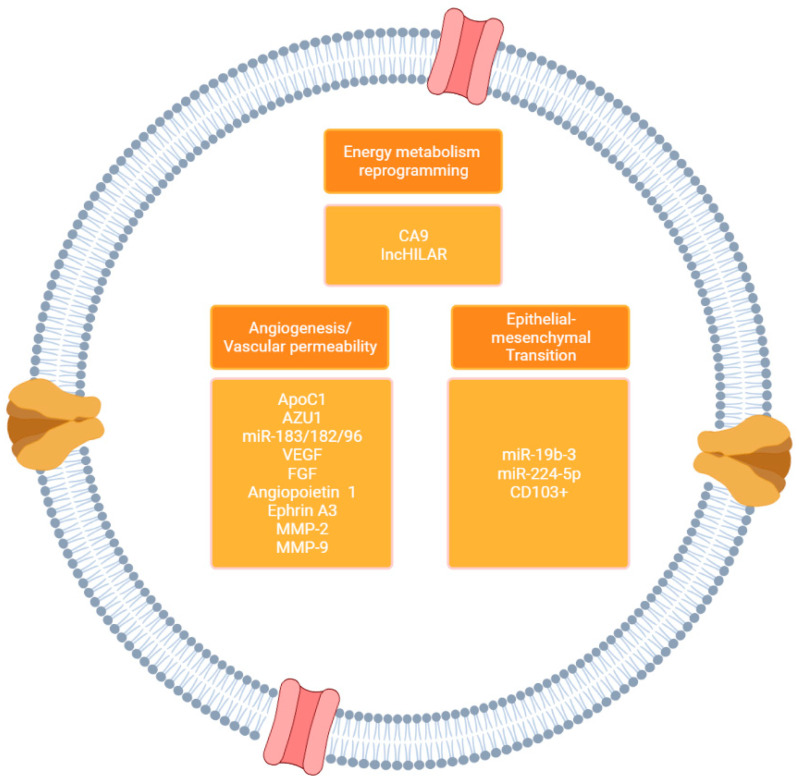
Relevant EV Cargo in Kidney Cancer. Renal cancer cells must integrate three critical processes towards establishing the pre-metastatic niche: 1. Angiogenesis/Vascular permeability, 2. Epithelial-mesenchymal transition, and 3. Energy metabolism reprogramming.

With respect to mRNA, studies have reported that undifferentiated tumor cells and CSCs release EVs containing multiple pro-angiogenic mRNAs, including vascular endothelial growth factor (VEGF), FGF, angiopoietin 1, ephrin A3, MMP-2, and MMP-9 [121]. These mRNAs play a crucial role in promoting endothelial cells’ growth, invasion, and survival, which ultimately support the progression of tumors and angiogenesis (Figure 3). Regulation of vascular endothelial cell proliferation and, consequently, angiogenesis are primarily governed by VEGF [160]. Exosomes isolated from human umbilical vein endothelial cells (HUVECs) were found to induce upregulated expression of VEGF in RCC, which correlated with the downregulation of hepatocyte cell adhesion molecule (HepCAM), a crucial tumor suppression gene that acts by inhibiting tumor angiogenesis [160]. Chen et al. described the regulatory influence of membrane-type MMPs, MT1-MMP and MT2-MMP, on the activation of MMP-2. MMP-2 is considered a necessary step in basement membrane degradation for cancer invasion [161]. However, in this study, the authors demonstrated that the activation of MMP-2 by MT2-MMP might enhance cell invasion and adhesion, leading to renal cancer progression. In a separate investigation, De Palma et al. affirmed that there were abnormal levels of mRNA in urine, specifically glutathione s-transferase alpha 1 (GSTA1), CCAAT enhancer binding protein alpha (CEBPA), and pterin-4 alpha-carbinolamine dehydratase 1 (PCBD1) [162]. Furthermore, after nephrectomy, the mRNA levels of these three genes returned to normal after one month, potentially indicating that the expression of these mRNA is related to the tumoral load. These findings demonstrate that the levels of mRNA in urinary EVs can potentially serve as molecular markers for RCC diagnosis. 

In hypoxic environments, RCC tumor cells secrete EVS containing/expressing Carbonic Anhydrase 9 (CA9) [163,164]. CA9 is a protein coding gene for CA9/CAIX transmembrane protein, a protein that is known to be over-expressed in VHL-mutated ccRCC and hypoxic solid tumors and that regulates intracellular pH and migration of endothelial cells [164,165]. Using in vivo angiogenesis assays, Horie et al. showed that after the uptake of CA9 exosomes, promotion of migration and tube formation was seen, as well as expression of MMP-2, which confers enhanced vascular proliferation capability [166]. Increasing evidence demonstrates how the TME has a strong correlation between the development of extravesicular cargo in relation to cell migration and the establishment of PMN [78,80] (Figure 3).

Among the different existing types of EV cargo, lncRNAs are a larger alternative to mRNA and miRNA, but they execute the same function of regulating key genes involved in the cancer progression and activation of oncogenic pathways and suppressing expression of tumor suppressors, thereby promoting tumor growth, invasion, and metastasis [167]. lncRNA are defined as RNAs longer than 200 nucleotides that are not translated into functional proteins and that fulfill regulatory roles during translation and transcription [168]. Another study described an upregulation of lncRNA in RCC and its association with hypoxia [168], supporting the association of hypoxia-induced lncRNA with renal cell carcinoma (lncHILAR). lncRNA was found to be a key modulator of hypoxic pathways, connecting with the miR-613/206/1-1-3p/Jagged1/Notch/CXCR4 axis to regulate the invasion and metastasis of RCC cells in hypoxic TME. These findings provide new insights into RCC progression and therapeutic approaches to impair clinical disease progression. 

The primary stromal components found in the tumor microenvironment of ccRCC are cancer-associated fibroblasts (CAFs) [169], which have a strong association with tumor progression. It was recently demonstrated that miR-224-5p was enriched in CAFS-derived exosomes in PMN, and after upregulation of miR-224-5p, there was a significant increase in the number of ccRCC cells undergoing migration and invasion promoting the progression of ccRCC [170]. Wang et al. examined the effect of EVs from CSCs in ccRCC on the advancement of EMT and the occurrence of lung metastases [171]. They found that miRNA (miR-19b-3) transported by EV mediated initiation of EMT after proving that the knockdown of miR-19b-3 resulted in downregulation of N-cadherin, Vimentin, and Twist, key molecules in charge of regulating migration, invasion, and adhesion of tumor cells. Moreover, they found that EVs derived from CSCs of metastatic cells had stronger fusion and contained a high proportion of CD103+ exosomes. This study concluded that CD103+ has a potent regulatory effect on EMT through the guidance of CSC exosomes to target distant organs and other cancer cells, assigning the higher metastatic capacity of ccRCC to lungs and pointing to CD103+ exosomes as a potential metastatic diagnostic biomarker [171]. Molecular metabolites transferred to neighboring cancer cells through EVs can affect the metabolism of the recipient cells in a way that promotes the progression of cancer [172]. Fu et al. found a shift in amino acid metabolism in ccRCC [173]. This study provided evidence of glutamine exhaustion, inducing secretion of IL-23 by macrophages via activation of the HIF1α pathway. IL-23 stimulates the activation of regulatory T cells (Treg) and enhances the production of IL-10 and TGF-β, leading to the suppression of T-cell cytotoxic activity and enabling immune evasion of tumor cells, highlighting the role of amino acid metabolism in immunosuppression [173] (Figure 3).

### 3.5. Monitoring Therapeutic Response in Renal Cancer Treatment

Extracellular vesicles and cfDNA assays have been studied as promising biomarkers to monitor or predict treatment responses in RCC. For non-metastatic RCC, the mainstay of therapy is nephrectomy with consideration of adjuvant systemic therapy for a fixed duration to prevent the recurrence of the disease. However, prediction of the surgical and clinical outcomes (i.e., recurrence) remains challenging. Current methods utilize primary clinical and imaging assessment. In patients with ccRCC, cfDNA levels decrease following surgery and remain low in patients without recurrence, whereas those with tumor recurrence showed a rise in cfDNA levels [174]. Moreover, the levels of ctDNA in an RCC patient who underwent nephrectomy became undetectable after surgery, increasing with disease progression [175]. Measuring somatic mutations in cfDNA in patients with non-metastatic ccRCC who underwent nephrectomy showed a decrease of less than 0.1% post-surgery [176]. 

For metastatic disease (mRCC), the primary treatment is systemic therapy with immune checkpoint inhibitors (ICI) and/or tyrosine kinase inhibitors (TKI) targeting vascular endothelial growth factor revectors (VEGFR) [122]. There is evolving data on the use of debulking surgery in metastatic disease that is incorporated into regimens including systemic therapy [122]. Kim et al. also included in their analysis that some patients with mccRCC who underwent non-curative cytoreductive surgery and demonstrated a decrease in the number of mutations after surgery in most cases [176]. For systemic therapies, lncRNA packaged exosomes have been demonstrated to be associated with VEGFK-TKI-resistant RCC cells, suggesting a possible use of exosomes to determine if a patient is developing treatment resistance [67,177]. This is attributed to the decreased expression of miR-549a in TKI-resistant cells and exosomes, which in turn upregulates HIF1α in endothelial cells. The reduction in the nuclear export of pre-miR-549a, mediated by the angiogenic VEGFR2-ERK-XPO5 pathway, and the diminished enrichment of mature miR-549a in the cytoplasm collectively promote HIF1α expression in RCC. Consequently, this leads to the increased secretion of VEGF and further activation of VEGFR2, thereby creating a feedback loop. Taken together, the findings suggest that miR-549a plays a critical role in the metastasis of ccRCC and has potential as a blood-based biomarker for detecting metastasis. Additionally, it could serve as a novel therapeutic agent to inhibit TKI-resistance [178]. A clinical study of 23 patients treated with VEGF TKIs involved serial collection of cfDNA ranging from 1 to 24 weeks of treatment. Two patients had a complete response, while two patients had a partial response with elevated levels prior to treatment. Seven patients experienced progressive disease and showed variability in pre- and post-treatment cfDNA levels [179]. Similar outcomes are found in patients treated with ICI. In a study of three patients who received ipilimumab and nivolumab, two had partial responses with ctDNA levels decreasing 1 month after the start of therapy [176]. Another clinical study of nine patients treated with nivolumab with or without ipilimumab showed that a decrease in the mutant allele frequency of ctDNA after therapy was associated with progression-free survival [180]. This evidence supports the utilization of cfDNA technologies as a part of longitudinal serial assessments in patients with RCC to determine treatment response to systemic therapy. 

In renal cancer, the study of EVs continues to be limited. The utilization of EVs as biomarkers for RCC diagnosis and prognosis presents challenges versus traditional markers, imaging methods, and pathological diagnosis. Current extraction technology and related costs hinder clinical use of EVs carrying cargo. There is still a need for high-throughput EV extraction, uniform cargo analysis standards, and bigger cohorts that can be generalized despite the molecular diversity that exists in RCC. Still, EVs’ major clinical application is in prognosis, metastatic risk stratification, and treatment guidance.

However, we consider that investigating CD103+ exosomes in kidney cancer, known for their robust EMT regulatory impact, holds promise as a novel strategy for treating metastatic CCRCC patients. Moreover, miR-19b-3p within exosomes could serve as a therapeutic target to formulate innovative approaches in thwarting CCRCC metastasis [171]. Similarly, the study of ctDNA in renal cancer treatment response has shown potential in clinical applications and in assessing quantitative alterations post-surgical intervention and immunotherapy [175,176].

## 4. Limitations

It is crucial to note that assigning a particular function to EVs as a whole or to specific EV subtypes demands more than just a basic description of their function in an unrefined, possibly contaminated, and mixed setup. It is known that asserting that exosomes possess exceptional and distinct activities is challenging to substantiate through experiments. This difficulty arises due to our current restricted understanding of the precise molecular processes governing their formation and release, especially compared to other EVs with similar biophysical properties. 

We recognize there are limitations associated with the use of EVs as non-invasive biomarkers. While EVs are feasibly collected from body fluids and typically are protected long enough for proper analysis, techniques for consistently capturing a quantity necessary for prognostic information pose a challenge [181]. Once samples are collected, there are no standardized methods for classifying, isolating, and characterizing exosomes, and similar-appearing materials like lipoproteins may contaminate samples or skew results [36,182]. Results of miRNA analysis from semen samples were compared to the standard ultracentrifugation technique. The expression profile of the altered semen EV-miRNAs in prostate cancer varied depending on EV isolation method applied [183]. This is possibly due to different extraction techniques yielding different proportions of semen EV subtypes [183]. These issues limit our understanding of the value of non-standardized assays and reliable comparison across studies, even if conducted in patients in similar disease settings [41,184]. 

In the area of EV research, even though many advances have been made in the last decade, there is an effort, such as the MISEV2018, to standardize how studies related to EVs are approached [6]. This guide proposes a way to differentiate the quality of the collected material depending on the specificity and the techniques used to isolate said material. It classifies EV methods as (a) high recovery, low specificity, (b) intermediate recovery, intermediate specificity, (c) low recovery, high specificity, and (d) high recovery and high specificity. It also explains that the source of EVs and the EV preparation must be described quantitatively and that no perfect method exists. Common quantification methods required for research in this field include protein, particle, and lipid assessments, including ratios of proteins and lipids to ensure purity and measurement reliability. Lastly, the guideline makes thorough recommendations about EV-associated function, highlighting the need to show that the function occurs independently of cell contact and differs from soluble factors. Also, it recommends that proving specificity for exosomes is cautioned against due to challenges if attempted; rigorous controls using proposed tools within their guidelines are needed to assess effects on other EVs, non-EV secretion, and cell physiology. Suggesting a universally applicable technique for all EV types is complex. Hence, we propose tailoring adherence to these guidelines based on charge molecule type, enhancing future investigative quality and standardization.

## 5. Future Directions

Despite the limitations surrounding the analysis of EVs, it is important to recognize a high potential for future development of EVs for advanced prostate and kidney cancers. Liquid biopsy analysis will serve as a beneficial, non-invasive tool in diagnosis, staging, and prognosis of these cancers, particularly in stratifying low- and high-risk patients in order to prevent over-diagnosis and overtreatment. Further techniques should be developed to standardize the collection and isolation of EVs, as this will bolster the efficacy of this methodology in becoming part of the standard guidelines for clinical decision-making for the management of cancer patients.

The rise in novel nanotechnologies has greatly improved the reproducibility of EV isolation and downstream computational analysis, consolidating the fundamentals of future EV research [185]. Consequently, given the proper biomarkers, EV-based liquid biopsy could serve as the new non-invasive alternative for clinical diagnosis and real-time disease monitoring [185]. Accumulating research has provided glimpses of both the transcriptomic and proteomic landscape of EVs derived from the biofluids of prostate [2,95,186,187,188,189] and renal cancer patients [190,191]. Specifically, in the case of prostate cancer, FABP5 has already demonstrated prospects in multiple studies as a urine EV marker that could effectively distinguish patients with Gleason score ≥ 7 [192,193,194]. Various miRNA EV markers, for example, miR-34 and miR-21, are also associated with different types of prostate cancer therapy resistance [55,195,196]. Likewise, in kidney cancer, De Palma et al. has identified three transcriptomic markers, including GSTA1, DCEBPA, and PCBD1, that are down regulated in urine EVs of ccRCC patients [162]. Overexpression of unique miRNA signatures, for instance, miR-224, miR-210, miR-1233, and miR-15a, are also observed in serum EVs of RCC patients [71]. While the majority of these studies still remain at translational level, cases of FDA-approved clinical trials and therapies involving EVs have multiplied since 2019. Similarly, there has been a rapid growth in a number of tissue-specific biomarkers found across different biofluids [7,31,197,198,199,200,201,202,203,204,205]. As our understanding of the EV biology and cargo delivery advances, uncovering reliable EV-based markers specific to different stages of progression in the biofluids and non-invasive liquid biopsy assays with higher accessibility will eventually supplant conventional screening methods, impacting patient therapeutic management.

## 6. Conclusions

Here, we delineate the role of EVs in prostate and renal cancers. We discuss in detail the EV biogenesis, extracellular release, cargo (DNA/RNA/protein), and their potential role in cellular crosstalk and reprogramming the tumor microenvironment. We deliberately detach from the generalized role of EVs in all cancers but argue their potential contribution as to how EVs may have both the pathogenic and therapeutic function in prostate and renal cancer subtypes. Finally, we present the role of liquid biopsy in kidney and prostate cancer prognosis and monitoring therapeutic response in cancer treatment. Overall, EVs carry proteo-transcriptomic cargo from their cancer cell of origin [2] and play a role in reprogramming the tumor microenvironment [206]. Yet, with accumulating evidence, we are now learning that EVs are heterogenous populations of micro- and nano-sized dimensions released by most cells and each subpopulation may have a distinct cargo and function [1]. As novel technologies and methodologies are developed, we foresee a canopy of extracellular vesicles and particles, which may have distinct functions in the pathogenesis of various diseases, including prostate and renal cancers.

## Figures and Tables

**Table 1:** A detailed list of prostate and renal cancer derived EV markers.

EV Markers	Source	Isolation Methods	Comparison	Application	Ref.
**Prostate Cancer**					
NKX3-1, BRCA1, MXD4, CYLD, IRF1 ESR1, SMYD3, FOXO3, HAS2	Serum, Urine	2000× *g* (30′ at 4 °C) + 10,000× *g* (45′ at 4 °C) + UC	Pre- vs. Post-prostatectomy	Diagnosis	[26]
miR-142-3p, miR-142-5p, miR-223-3p	Semen	1600× *g* (10′) + 16,000× *g* (10′ at 4 °C) + MF (0.22 μm) + UC	PCa vs. BPH	Diagnosis	[53]
miR-342-3p, miR-374b-5p			PCa (GS ≥ 7) vs. Control (PSA ≥ 4 ng/mL)	Diagnosis	
miR-423-3p, miR-320a, miR-99a-5p, miR-320d, miR-320b, miR-150-5p	Plasma	2000× *g* (10′) + Total Exosome Isolation Kit (from plasma) (Invitrogen™ Thermo Fisher Scientific, Waltham, MA, USA),	CRPC vs. treated non-CRPC	Prognosis	[54]
miR-34a	22RV1, DU145, PC3	10,000× *g* (30′ at 4 °C) + MF (0.22 μm) + UC	Docetaxel-resistant vs. unexposed	Prognosis	[55]
CD44	PC3	1800× *g* (10′) + 16,500× g (20′) +UC	Docetaxel-resistant vs. unexposed	Prognosis	[56]
AR-V7	Plasma	10,000× *g* (30′) + ExoQuick^®^ (System Biosciences, CA, USA)	mCRPC vs PCa	Prognosis	[57]
miR-1290, miR-375	Plasma	12,300× *g* (5′) + ExoQuick^®^ (System Biosciences, CA, USA)	CRPC vs. ADT	Prognosis	[58]
TUBB3	Plasma	1500× *g* (15′) + exoEasy	mCRPC with abiraterone vs. mCRPC without	Prognosis	[59]
miR-375, miR-34a, miR-19b-3p, miR-30d-5p	Serum	2000× *g* (30′ at 4 °C) + 10K ultracel filter + 10,000× *g* (1 h)	CRPC-Adeno vs. CRPC-NED	Diagnosis	[60]
BRN4, BRN2	Serum	Total exosome isolation reagent (Thermo Fisher scientific^TM^, Waltham, MA, USA)	CRPC-Adeno vs. CRPC-NED	Diagnosis	[61]
**Renal Cancer**					
lncARSR	Serum	100,000× *g* (70′ at 4 °C) + used Théry C et al. technique [62]	ccRCC vs. Healthy Controls	Diagnosis	[63]
Azurocidin	Serum	2000× *g* (30′) + Total exosome isolation (Thermo Fisher scientific^TM^, Waltham, MA, USA) for xenografted mice, EVSecond (GL columns TM for healthy controls)	ccRCC vs. Healthy Controls	Diagnosis	[64]
miR-126-3p and miR-449a	Urine	800× *g* (5′) + 2000× g (10′) + TRIzol Plus RNA Purification Kit (Life Technologies^TM^, Carlsbad, CA, USA)	ccRCC vs. Healthy Controls	Diagnosis	[65]
miR-30c-5p	Urine	miRNeasy	ccRCC vs. Healthy Controls	Diagnosis	[66]
miR-210, miR-1233	Serum	1200× *g* (10′ at 4 °C) + Total exosome isolation reagent (Invitrogen™, Thermo Fisher scientific^TM^, Waltham, MA, USA)	ccRCC vs. Healthy Controls	Diagnosis	[67]
miR-210	Serum	1000× *g* (10′ at 4 °C) +Total exosome isolation reagent (Invitrogen™, Thermo Fisher scientific^TM^, Waltham, MA, USA)	ccRCC vs. Healthy Controls	Diagnosis	[68]
miR-21-5p, miR-150-5p, miR-145-5p, and miR-146a-5p	Serum	3000× *g* (10′ at 4 °C) + TRIzolTM LS Reagent (Invitrogen, Thermo Fisher scientific^TM^, Waltham, MA, USA)	RCC vs. Healthy Controls	Diagnosis	[69]
miR-21-5p	Plasma	2500× *g* (30′ at 4 °C) + MagMAX mirVana Total RNA Isolation kit (Thermo Fisher™, Waltham, MA, USA)	RCC vs. Healthy Controls	Diagnosis	[70,71]
miR-15a	Urine	mirVana™ miRNA Isolation Kit (Applied Biosystems, Thermo Fisher™, Waltham, MA, USA)	RCC vs. Benign Renal Tumors	Diagnosis	[72]
PTRF	Urine	100,000× *g* (70′ at 4 °C) used Thery C et al. technique [62]	ccRCC vs. Healthy Controls	Diagnosis	[73]
NME2, AAMP, CAPNS1, VAMP8, and MYL12B	Urine	4300× *g* (30′ at 20 °C) + ExoQuick-TC PLUS Exosome Purification Kit (System Biosciences, Palo Alto, CA, USA)	stage I ccRCC vs. Healthy Controls	Diagnosis	[74,75]
cfDNA methylation	Serum	1800× *g* (10′) + QIAamp Ultrasens Virus Kit (Qiagen, Venlo, The Netherlands)	RCC vs. Healthy Controls	Diagnosis	[76]
GSTA1, CEBPA, PCBD1	Urine	300× *g* (10′) +17,000× *g* (20′) + 118,000× g (70′) + miRCURY RNA isolation kit (Exiqon, Vedbaek, Denmark)	ccRCC vs. Healthy Controls	Prognosis	[77]
miR-224-5p	Tissue	2500× *g* (10′) + The Hieff™	ccRCC	Progression	[78]
miR-19b-3	Tissue	100,000× *g* (90′) used Liu et al. Technique [79]	ccRCC	Progression	[80]

## Data Availability

The data used in this manuscript is derived from The Cancer Genome Atlas Prostate Adenocarcinoma (TCGA-PRAD) data collection.

## Abbreviations

ADT	Androgen deprivation therapy
AML	Angiomyolipoma
ApoC1	Apolipoprotein C1
AR	Androgen receptor
AR-FL	Full-length androgen receptor
ARPIs	Androgen receptor pathway inhibitors
AUC	Area under the curve
BCL-2	B-cell lymphoma 2
BPH	Benign prostate hypertrophy
CAFs	Cancer-associated fibroblasts
ccRCC	Clear cell renal cell carcinoma
cfDNA	Circulating free DNA
CRPC	Castration-resistant prostate cancer
CRPC-Adeno	Castration-resistant prostate cancer adenocarcinoma
CRPC-NE	Castration-resistant prostate cancer neuroendocrine
CSCs	Cancer stem cells
CTCs	Circulating tumor cells
ctDNA	Circulating tumor DNA
ctRNA	Circulating tumor RNA
DRE	Digital rectal examination
EMT	Epithelial-mesenchymal transition
ESCRT	Endosomal sorting complexes required for transport
EVs	Extracellular vesicles
GHRH	Growth hormone-releasing hormone
GS	Gleason Score
HDL	High-density lipoprotein
HRS	Hepatocyte growth factor regulated tyrosine kinase substrate
ICI	Immune checkpoint inhibitors
ILVs	Intraluminal vesicles
LNARSR	lncRNA in serum
MF	Microfiltration
MiPS	Mi-Prostate Score
MVBs	Multivesicular bodies
NEPC	Neuroendocrine prostate cancer
OS	overall survival
PCA3	Prostate cancer antigen 3
PFS	Progression-free survival
PHI	Prostate Health Index
PLP	Proteolipid protein
PMN	Pre-metastatic niche
PSA	Prostate-specific antigen
PTRF	Polymerase 1 and transcriptase release factor
RCC	Renal cell carcinoma
reg	Regulatory T cells
ROS	Reactive oxygen species
smRCs	small RNA clusters
SNAREs	SNAP receptors
TCGA-PRAD	The Cancer Genome Atlas Prostate Adenocarcinoma Cohort
tdEVs	Tumor-derived EVs
TFs	Transcription factors
TKI	Tyrosine kinase inhibitor
TME	Tumor microenvironment
TRUS	Transrectal ultrasonography
TSE	Tumor-secreted exosome
TSF	Tumor-secreted factor
TSP1	Thrombospondin 1
TWEAK	Tumor necrosis factor-like weak inducer of apoptosis
UC	Ultracentrifugation
VEGFR	Vascular endothelial growth factor revectors
VLDL	Very low-density lipoprotein
